# Terpinen-4-ol Induces Ferroptosis of Glioma Cells via Downregulating JUN Proto-Oncogene

**DOI:** 10.3390/molecules28124643

**Published:** 2023-06-08

**Authors:** Wenpeng Cao, Yumei Li, Zhirui Zeng, Shan Lei

**Affiliations:** 1 Department of Anatomy, School of Basic Medicine, Guizhou Medical University, Guiyang 550025, China; caowenpeng@gmc.edu.cn (W.C.); liyumei@gmc.edu.cn (Y.L.); 2 Department of Physiology, School of Basic Medicine, Guizhou Medical University, Guiyang 550025, China

**Keywords:** terpinen-4-ol, glioma, ferroptosis, proliferation, JUN

## Abstract

According to previous research, turmeric seeds exhibit anti-inflammatory, anti-malignancy, and anti-aging properties due to an abundance of terpinen-4-ol (T4O). Although it is still unclear how T4O works on glioma cells, limited data exist regarding its specific effects. In order to determine whether or not glioma cell lines U251, U87, and LN229 are viable, CCK8 was used as an assay and a colony formation assay was performed using different concentrations of T4O (0, 1, 2, and 4 μM). The effect of T4O on the proliferation of glioma cell line U251 was detected through the subcutaneous implantation of the tumor model. Through high-throughput sequencing, a bioinformatic analysis, and real-time quantitative polymerase chain reactions, we identified the key signaling pathways and targets of T4O. Finally, for the measurement of the cellular ferroptosis levels, we examined the relationship between T4O, ferroptosis, and JUN and the malignant biological properties of glioma cells. T4O significantly inhibited glioma cell growth and colony formation and induced ferroptosis in the glioma cells. T4O inhibited the subcutaneous tumor proliferation of the glioma cells in vivo. T4O suppressed JUN transcription and significantly reduced its expression in the glioma cells. The T4O treatment inhibited GPX4 transcription through JUN. The overexpression of JUN suppressed ferroptosis in the cells rescued through T4O treatment. Taken together, our data suggest that the natural product T4O exerts its anti-cancer effects by inducing JUN/GPX4-dependent ferroptosis and inhibiting cell proliferation, and T4O will hope-fully serve as a prospective compound for glioma treatment.

## 1. Introduction

Among the primary brain tumors, gliomas are the most common. They are caused by the interaction of multiple factors and have biological characteristics of malignancy, such as high aggressiveness, abnormal metabolism, and angiogenesis [1]. Approximately fifteen months is the median survival time of patients with glioblastomas, with a high recurrence rate [2]. Surgical resection is the main treatment method, and the post-operative adjuvant grading of radiotherapy combined with chemotherapy also plays a very important role [3]. At present, the first-line chemotherapy drug temozolomide has obvious efficacy but is limited by drug resistance [4]. Therefore, the development of new drugs to treat gliomas is of considerable clinical significance.

Medicinal plants are a huge source for drug discovery, and numerous studies have shown that plant extracts have anti-proliferative or anti-tumor effects on tumor cells [5]. Terpinen-4-ol (T4O) has anti-tumor, anti-bacterial, anti-fungal, anti-platelet, anti-oxidation, anti-senile dementia, and anti-metabolic syndrome properties [6]. Ken et al. reported that T4O inhibits colorectal cancer growth by increasing the levels of reactive oxygen species [7]. Furthermore, Cao et al. have shown that terpine-4-ol inhibits the proliferation of pancreatic cancer cells by downregulating the Rho-associated coiled-coil protein kinase 2 [6]. However, the effects of T4O and its underlying mechanisms on gliomas have not yet been clarified.

As a transcription factor, c-Jun, also called activator protein 1, plays a key role in the regulation of neuronal death and regeneration [8]. The JUN protein is believed to play a significant role in tumor development and occurrence, mainly regulating tumor cell survival and apoptosis [9,10,11]. The cells of glioblastomas were effectively killed by JUN-ELP-KLAK as demonstrated by Sarangthem et al. [12]. Bhardwaj et al. indicate that the IL-13/IL-13Rα2 axis can mediate signal transduction in situ via the JUN pathway in glioblastoma multiformes [13]. Dang et al. reported that the tumor-suppressing role of miR-218 is caused by the blockage of the TNC/AKT/JUN/TGFβ1-positive feedback loop in gliomas [14]. There have been some inhibitors identified to date that target JUN, such as T-5224 and E3330, that exhibit distinct anti-tumor properties [15,16]. As a result, JUN inhibition could be considered a potential chemotherapeutic target for gliomas.

The purpose of the present study was to identify the specific effects and key molecular mechanisms of T4O in gliomas, which may aid in the treatment of this disease. The T4O treatment reduced JUN’s activity and induced ferroptosis in the cells, inhibiting the proliferation of the glioma cells. As a result of these findings, T4O may provide a promising treatment option for gliomas arising from its novel mechanism of anti-GBM activity.

## 2. Results

### 2.1. T4O Inhibited Glioma Cell Proliferation and Colony Formation

**Figure 1:**
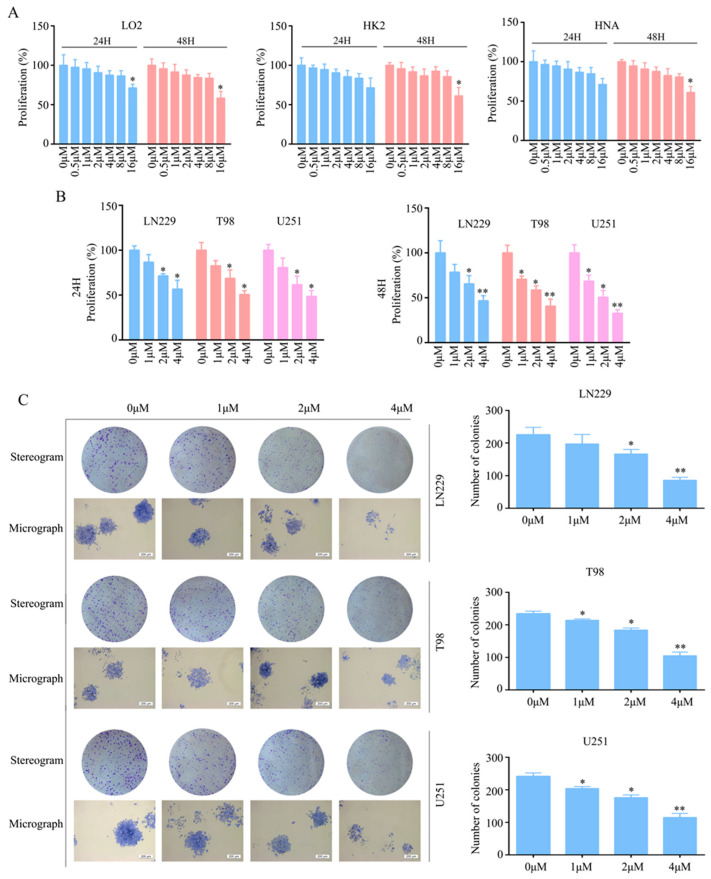
T4O inhibition of glioma cell proliferation in vitro. (**A**) Cell proliferation rate of LO2, HK2, and HNA cells treated with different concentrations (0, 0.5, 1, 2, 4, 8, and 16 μM) of T4O at 24 and 48 h as determined using the CCK-8 assay. (**B**) Results of U251, T98, and LN229 cells treated with different concentrations (0, 1, 2, and 4 μM) of T4O; CCK-8 assay was used to detect the proliferation in each group. (**C**) Results of colony formation assay used to detect the colony formation of glioma cells which were treated with different concentrations (0, 1, 2, and 4 μM) of T4O. * represents *p* < 0.05; ** represents *p* < 0.01. *n* = 3. The control group was used for comparison. Data are shown as mean ± SD.

The treatment of LO2, HK2 and HNA cells with 0, 0.5, 1, 2, 4, 8 and 16 μM of T4O revealed that 0, 1, 2, and 4 μM T4O was not cytotoxic to the LO2, HK2 and HNA cells at both 24 and 48 h (Figure 1A). To rule out non-specific cytotoxicity, follow-up experiments were performed using 0, 1, 2, and 4 μM T4O. The glioma cell lines (LN229, T98, and U251) were treated with 0, 1, 2, and 4 μM T4O. According to the CCK-8 assay, T4O markedly inhibited the cell proliferation of LN229, T98, and U251 at 24 and 48 h (Figure 1B). Moreover, the colony formation assays revealed that the colonies were fewer, smaller, and incompact in the T4O treatment group compared with those in the DMSO treatment group (Figure 1C).

### 2.2. T4O Suppressed the Proliferation of Glioma Cells In Vivo

**Figure 2:**
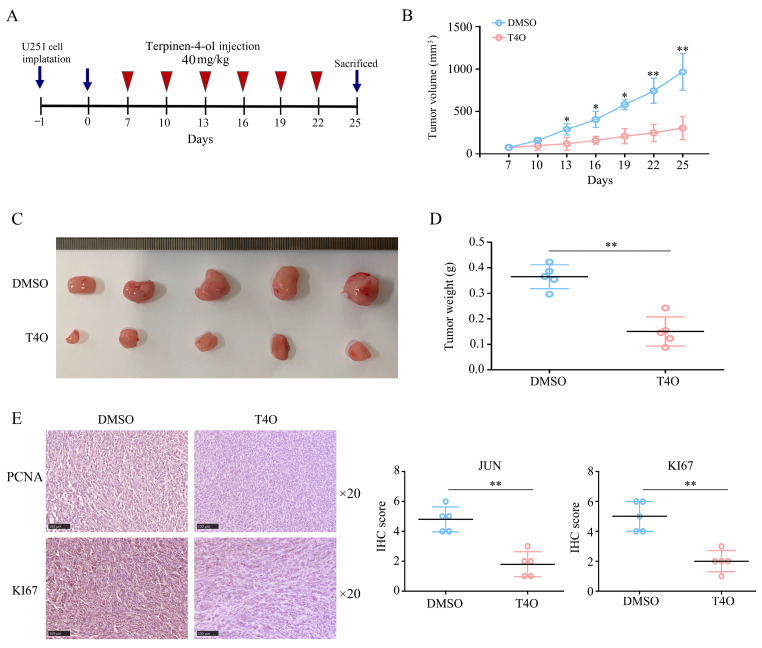
T4O repression of the proliferative rate of U251 cells in vivo. (**A**) Model diagram of animal experiments. (**B**,**C**) Proliferation of tumor tissues treated with DMSO and T4O. (**D**) Tumor weight of tumor tissues treated with DMSO and T4O. (**E**) Expression of PCNA and KI67 in the tumor tissues treated with DMSO and T4O. * represents *p* < 0.05; ** represents *p* < 0.01. *n* = 5. The control group was used for comparison. Data are shown as mean ± SD.

The T4O treatment was assessed in vivo by subcutaneously injecting U251 cells into BALB/c nude mice. The DMSO and T4O (40 mg/kg) groups were randomly divided. In the DMSO group, tumor growth was rapid, and T4O significantly inhibited tumor growth (Figure 2A–C) as well as reducing tumor weight (Figure 2D). Furthermore, the T4O treatment resulted in lower levels of KI67 and PCNA protein expression in the tumor tissues compared with the DMSO treatment (Figure 2E). The results showed that T4O had an obvious anti-tumor effect in vivo.

### 2.3. JUN Was Identified as a Key Target of T4O

**Figure 3:**
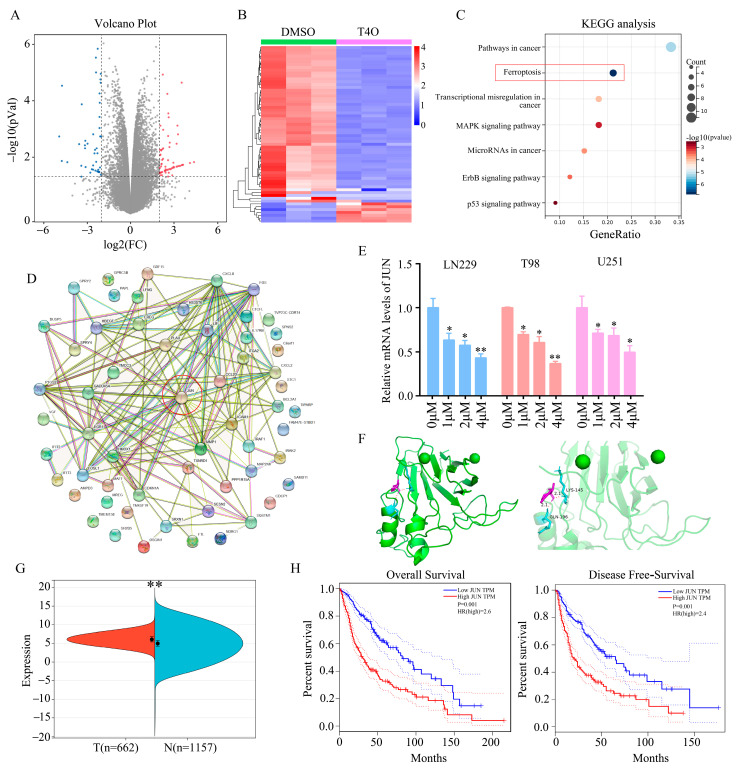
JUN as the key target of T4O. (**A**,**B**) Identified differentially expressed genes in glioma cells treated with DMSO and T4O, Red represents up genes; blue represents down genes; gray represents non-significant genes. (**C**) Results of KEGG analysis performed to determine the pathways in which differentially expressed genes were enriched. (**D**) Results of protein–protein interaction network analysis performed for differentially expressed genes. JUN had strong relationship with other proteins encoded by differentially expressed genes. (**E**) Results of RT-qPCR performed to analyze the mRNA levels of JUN in glioma cells treated with different concentrations (0, 1, 2, and 4 μM) of T4O. (**F**) Binding mode of T4O with JUN and 3D illustration of the details of the interaction. Purple represents T4O; green represents the JUN protein. (**G**) JUN expression in gliomas and adjacent tissues according to the data from TCGA and GTEx databases. (**H**) KM plot showing the OS and DFS of patients with low and high JUN expression according to data from the TCGA database. * represents *p* < 0.05; ** represents *p* < 0.01. *n* = 3. The control group was used for comparison. Data are shown as mean ± SD.

As part of our investigation into the molecular mechanism of T4O in gliomas, high-throughput sequencing was performed on the U251 cells treated with T4O. We found 46 genes that were downregulated and 63 genes that were upregulated (Figure 3A,B). An analysis of the KEGG data revealed 109 differentially expressed genes that were significantly enriched in cancer-related pathways, ferroptosis, transcriptional misregulation in cancer, the MAPK signaling pathway, microRNAs in cancer, the ErbB pathway and the p53 signaling pathway (Figure 3C). An analysis of the PPI networks revealed that JUN was strongly linked to the proteins encoded by other differentially expressed genes (Figure 3D). RT-qPCR and Western blotting revealed that both the JUN mRNA and protein levels were elevated in the glioma cells treated with T4O (Figure 3E). Molecular docking technology was then used to analyze the binding model of T4O and the JUN protein. Ac-cording to the 3D drawing, T4O binds to GLN196 and LYS145 in the JUN protein in a stabilizing manner (Figure 3F). The JUN expression level in glioma tissues was found to be significantly higher compared to that in normal tissues in the TCGA and GTEx database data (Figure 3G). We found that elevated JUN expression was associated with lower OS and DFS rates in GBM patients (Figure 3H).

### 2.4. T4O Induced Glioma Cell Ferroptosis

**Figure 4:**
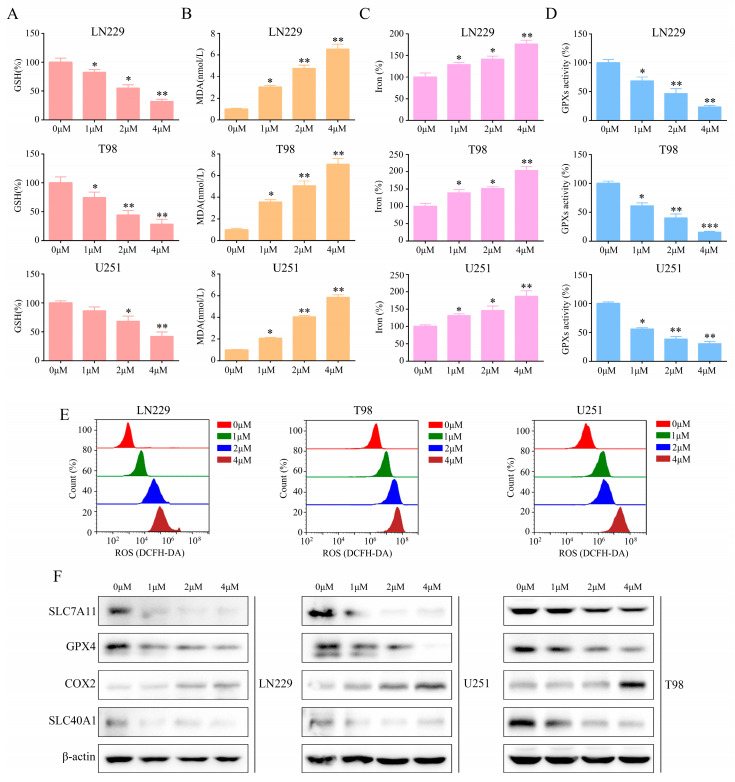
T4O inducement of ferroptosis in glioma cells in vitro. (**A**) Intracellular GSH levels in glioma cells treated with different concentrations (0, 1, 2, and 4 μM) of T4O. (**B**) Intracellular MDA levels in glioma cells treated with different concentrations (0, 1, 2, and 4 μM) of T4O. (**C**) Intracellular iron progression in glioma cells treated with different concentrations (0, 1, 2, and 4 μM) of T4O. (**D**) Intracellular GPX activities in glioma cells treated with different concentrations (0, 1, 2, and 4 μM) of T4O. (**E**) Results of U251, T98, and LN229 cells treated with different concentrations (0, 1, 2, and 4 μM) of T4O; the cellular ROS level was analyzed with a flow cytometer. (**F**) Results of U251, T98, and LN229 cells treated with different concentrations (0, 1, 2, and 4 μM) of T4O;the expression of ferroptosis-related proteins was detected through Western blotting. * represents *p* < 0.05; ** represents *p* < 0.01; *** represents *p* < 0.001. *n* = 3. The control group was used for comparison. Data are shown as mean ± SD.

Reactive oxygen species (ROS), lipid peroxidation, and glutathione depletion are hallmarks of ferroptosis [17]. Consequently, the T4O treatment of the glioma cells detected levels of ROS, glutathione (GSH), and malondialdehyde (MDA) as markers of oxidative stress. Ferroptosis-related events, such as glutathione depletion (Figure 4A), malondialdehyde production (Figure 4B), and elevated iron levels, occurred (Figure 4C). The T4O treatment of the glioma cells consistently exhibited low baseline GSH peroxidase (GPX) activity (Figure 4D). Furthermore, the glioma cells treated with T4O showed high baseline reactive oxygen species (ROS) levels (Figure 4E). The glioma cells treated with T4O showed a significant reduction in the expression of the negative regulators of ferroptosis, GPX4, COX2, SLC40A1, and SLC7A11 (Figure 4F). Together, these findings strongly suggest that T4O induces ferroptosis in glioma cells.

### 2.5. GPX4 Is a Downstream Gene of JUN

**Figure 5:**
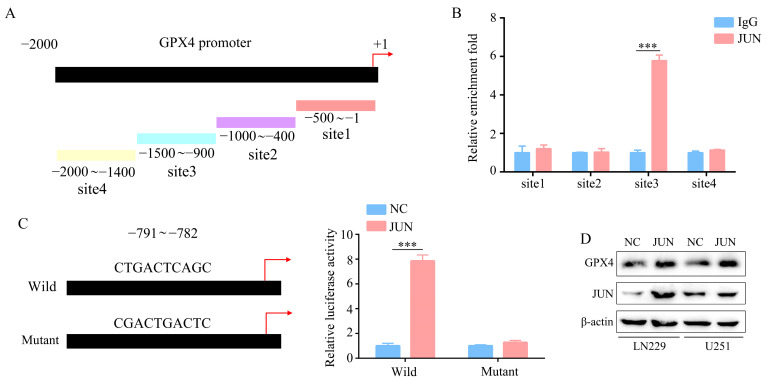
JUN regulation of the expression of GPX4 by binding to the GPX4 promoter. (**A**) GPX4 gene promoter region. (**B**) Results of the chromatin immunoprecipitation (ChIP) assay performed to identify which region functioned as the effective binding site of the GPX4 promoter region. (**C**) Results of the glioma cells transfected with either a full-length or truncated GPX4 promoter–pGL3 reporter vector and further cultured either with or without JUN plasmid. After 48 h, luciferase activity was measured using the dual-luciferase reporter assay system. (**D**) Results of Western blotting which detected the effect of JUN overexpression on the protein expression of GPX4. *** represents *p* < 0.001. *n* = 3. The control group was used for comparison. Data are shown as mean ± SD.

Figure 5A shows how the promoter region of the GPX4 gene is divided into four sections. According to the chromatin immunoprecipitation (ChIP) assay, endogenous JUN binds to the GPX4 promoter regions (−1400~−800) (Figure 5B). After the ChIP assay, we performed bioinformatics to predict the transcription factor JUN’s binding sites of the GPX4 gene promoter and found that JUN could bind to sites in the GPX4 gene promoter region (−791~−782). The wild-type or mutant sequences of the GPX4 promoter region (−791~−782) were established. The overexpression of JUN in the glioma cells significantly increased the luciferase activity of the wild-type sequences in the GPX4 promoter region. In contrast, the mutant sequences in the GPX4 promoter region did not exhibit increased luciferase activity as evidenced by the results (Figure 5C). Based on a Western blot analysis, enhanced transfection with a JUN plasmid increases GPX4 protein expression in glioma cells (Figure 5D).

### 2.6. Overexpression of JUN Attenuated the Inhibitory Effect of T4O on Glioma Cell Proliferation 

**Figure 6:**
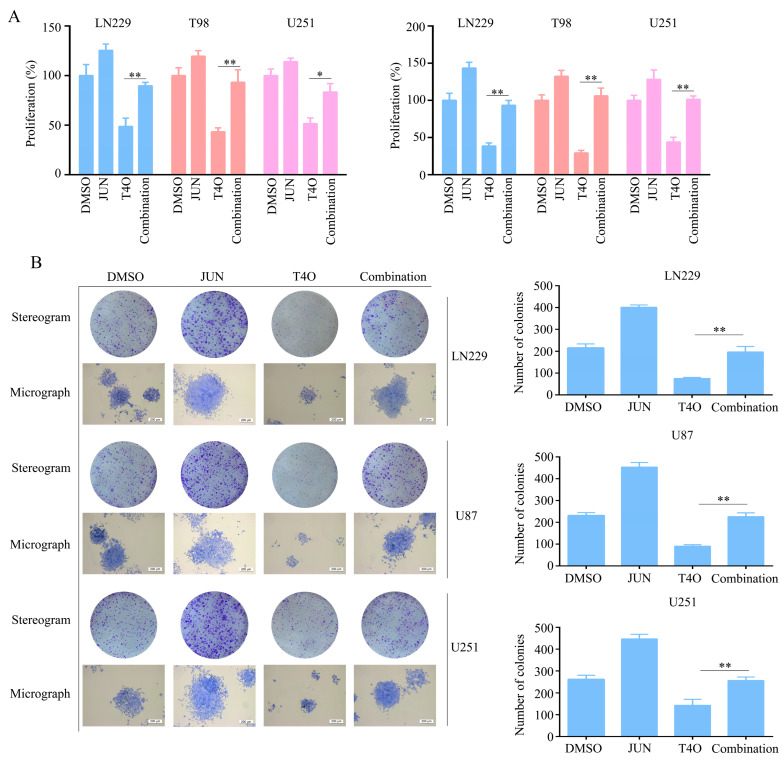
Reversal of inhibitory effects of T4O on proliferation of glioma cells due to overexpression of JUN; glioma cells were treated with DMSO, T4O, JUN plasmid and T4O + JUN plasmid, respectively. (**A**) Results of the use of CCK-8to detect the proliferative rate of glioma cells in each group. (**B**) Results of colony formation assay used to detect the colony formation of glioma cells in each group. * represents *p* < 0.05; ** represents *p* < 0.01. *n* = 3. The control group was used for comparison. Data are shown as mean ± SD.

As JUN has been associated with several types of cancer, we hypothesized that it might be involved in T4O-induced biological functions. Several plasmids containing JUN were transfected into the glioma cells before the T4O treatment. According to the CCK-8 assay, JUN overexpression alleviated both the 24 and 48 h suppression of glioma cell proliferation caused by T4O (Figure 6A). The overexpression of JUN alleviated the suppressive effect of T4O on colony formation in the glioma cells, according to the colony formation assays (Figure 6B). 

### 2.7. Overexpression of JUN Attenuated the Promotion of Ferroptosis by T4O in Glioma Cells

**Figure 7:**
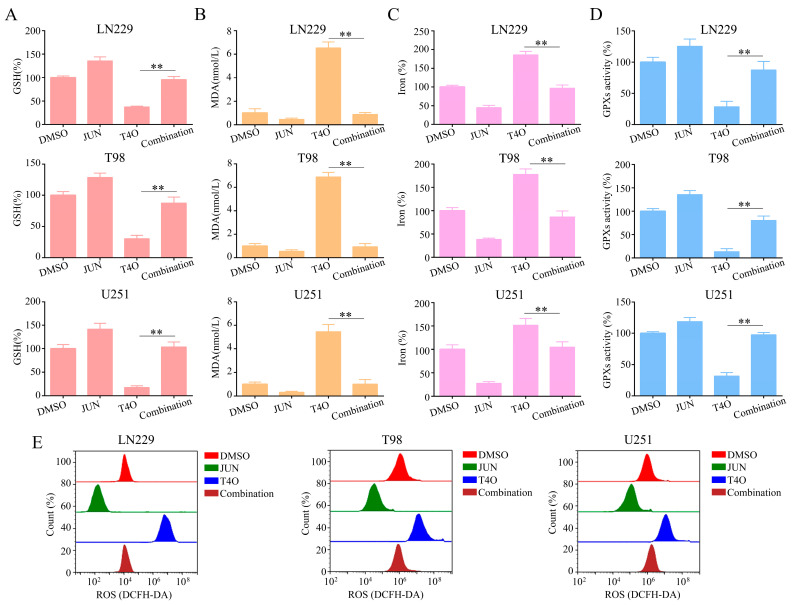
Reversal of the promotive effect of T4O on ferroptosis in glioma cells due to overexpression of JUN; lioma cells were treated with DMSO, T4O, JUN plasmid, and T4O + JUN plasmid, respectively. (**A**) Intracellular GSH levels in glioma cells in each group. (**B**) Intracellular MDA levels in glioma cells in each group. (**C**) Intracellular iron progression in glioma cells in each group. (**D**) Intracellular GPX activities in glioma cells in each group. (**E**) Cellular ROS level was analyzed with a flow cytometer in each group. ** represents *p* < 0.01. *n* = 3. The control group was used for comparison. Data are shown as mean ± SD.

Finally, several plasmids containing JUN were transfected into the glioma cells before the T4O treatment. There was an increase in ferroptosis-related events in the glioma cells with T4O treatment, including a decrease in GSH (Figure 7A), an increase in MDA production (Figure 7B), an elevated iron level (Figure 7C), and a decrease in GPX activity (Figure 7D) as well as high baseline reactive oxygen species (ROS) levels (Figure 7E); the overexpression of JUN attenuated the above effects.

## 3. Discussion

Over the past few years, TCM has attracted the attention of many researchers worldwide owing to its unique advantages, including its multiple pathways, multiple targets, low toxicity, and few side effects [18,19,20]. Turmeric, a traditional Chinese medicine, is rich in curcumin and turmeric volatile oil [21]. Studies have confirmed that curcumin and a variety of curcumin compounds promote the early apoptosis of glioma cells, inhibit their growth and migration, induce DNA damage, and have been identified as potential anti-glioma drugs [22,23]. For example, Majchrzak et al. showed that sodium butyrate increased the permeability of curcumin through the blood–brain barrier, restored the expression of Wnt/β-catenin pathway antagonist genes, and reduced the vitality of glioblastoma cells [24]. Liu et al. found that β-elemene enhanced the radiosensitivity and chemical sensitivity of glioblastoma cells by inhibiting ATM signaling pathways [25]. Majchrzak et al. confirmed that nomethoxylcurcumin prevents human glioblastoma multiforme cells from proliferating, migrating, and invading [26]. However, the inhibitory effect of terpinen-4-ol on gliomas has not yet been reported at home or abroad.

In the present study, through a series of functional experiments, we found that T4O significantly inhibited glioma cell proliferation and colony formation as well as inducing ferroptosis. This evidence demonstrated that T4O exhibits distinct anti-glioma effects. It has been suggested that T4O is a promising anti-glioma drug. RNA-seq was further used to investigate the molecular pattern and biological function of T4O in the glioma cells; 109 genes showed significant changes after treatment with T4O. Differential gene expression in ferroptosis was significantly enriched. It is interesting to note that, after treatment with T4O, the JUN levels were downregulated in the glioma cells. JUN might be associated with glioma survival prognoses, and patients who express high levels of gliomas have shorter OS and DFS. In addition to being a member of the basic leucine zipper transcription factor family, the JUN proto-oncogene belongs to the JUN family of immediate-early genes [27]. Regulatory proteins in the intracellular JUN family form homodimers or heterodimers with each other as well as with those in the FOS family, and they are closely related to the transcriptional regulation of many cytokines and growth factors in the cell [28,29]. Several malignant tumors express JUN, which is essential for regulating proliferation, apoptosis, and malignant transformation [30,31]. The mechanism by which JUN promotes ferroptosis in gliomas was identified. A binding site in JUN specifically interacts with the GPX4 promoter to promote GPX4 transcription.

There is strong evidence that ferroptosis plays a role in several pathological conditions, such as cancer, neurological degeneration, and ischemia–reperfusion injuries [32]. A variety of cancer types have been shown to be controlled by ferroptosis, including their initiation, development, invasion, metastasis, and therapeutic resistance [33]. A recent study has revealed that GPX4, a phospholipid hydroperoxide glutathione peroxidase, promotes ferroptosis, thereby promoting cancer growth [34]. This protein prevents membrane lipid peroxidation from causing cell death and maintains intracellular redox homeostasis [32]. In previous studies, GPX4 was shown to be highly expressed in gliomas and to be closely associated with their progression [35].

In the present study, we found that T4O can suppress glioma cell proliferation and induced ferroptosis; the JUN mRNA levels were reduced in the glioma cells following the T4O treatment. JUN specifically interacted with the GPX4 promoter and upregulated GPX4 expression. JUN overexpression markedly suppressed the effects of T4O on glioma cell proliferation and ferroptosis. These results indicated that JUN is involved in T4O-induced biological processes.

## 4. Materials and Methods

### 4.1. Cell Culture

The American Type Culture Collection (ATCC, Rockville, MD, USA) provided glioma cell lines U251, T98, and LN229 and LO2, HK2, and HNA. DMEM (Gibco, Grand Island, NY, USA) with 10% FBS (BI, Kibbutz Beit Haemek, Israel) was used for both of the cell cultures at 37 °C with 5% CO_2_. The terpinen-4-ol (Fengyao Tonghui Chemical, Wuhan, China) powder was prepared in DMSO to form a mother liquor at a concentration of 10 mol·L^−1^. The DMEM culture solution was diluted with various concentrations of the working solution. Shanghai Kei Lei Biological Technology Co., Ltd. (Shanghai, China) constructed JUN plasmids. The transfection of plasmids into cells was carried out using Lipofectamine Lipo2000 (Thermo Fisher Scientific, Waltham, MA, USA).

### 4.2. CCK-8 Assay

The cell suspension was prepared by digesting the glioma cells in the logarithmic growth phase with trypsin. In total, 3 × 10^3^ cells were seeded in each well of a 96-well plate, and six holes in each group were set in each group. In each well, 90 μL of serum-free DMEM and 10 μL of the CCK-8 solution were added following 24 and 48 h of T4O treatment. A 2 h incubator culture was measured using microplate readers at 450 nm.

### 4.3. Colony Formation Assay

A total of 1 × 10^3^ glioma cells were seeded in six-well plates and were treated with different concentrations (0, 1, 2, and 4 μM) of T4O. After culturing for 14 days, discarding the medium, the cell colonies were fixed with 4% paraformaldehyde. They were then washed with PBS and were stained for 30 min with 0.5% crystal violet solution. Thereafter, a stereogram and micrograph of the colony plates were obtained using a camera and optical microscope (magnification 40×), respectively.

### 4.4. Western Blot

A phenolmethylsulfonyl–fluoride-containing lysis buffer containing radioimmunoassay precipitation was used to extract the total protein from glioma cells (Servicebio, Wuhan, China). In order to determine the protein concentrations in the samples, bicinchoninic acid (BCA) protein assays were performed (Servicebio, Wuhan, China). Separating proteins with sodium dodecyl sulfate polyacrylamide gels (Thermo Fisher Scientific, Waltham, MA, USA) led to their transfer to polyvinylidene fluoride membranes. Using skimmed milk powder to block the membranes, primary antibodies containing SLC7A11 (Cat No. 26864-1-AP), COX2 (Cat No. 66351-1-Ig), GPX4 (Cat No. 67763-1-Ig), JUN (Cat No. 66313-1-Ig), and β-actin (Cat No. 81115-1-RR) were purchased from proteintech; SLC40A1 (Cat No. ab239583) was purchased from abcam, in a dilution of 1:1000, was added for 2 h at room temperature and kept overnight at 4 °C. In total, 2 h of incubation was conducted at room temperature with a secondary antibody (in a dilution of 1:2000) after washing three times. A high-sensitivity ECL exposure solution was added and developed using an imager. β-actin was used as the loading control to calculate the relative protein expression.

### 4.5. Subcutaneous Tumorigenesis Experiments

The Animal Center of Guizhou Medical University purchased female BALB/c nude mice. A cell suspension of a total of 100 μL of 2 × 10^6^ U251 cells was injected subcutaneously into the right armpit of the forelimb of each mouse. On day 7, tumor size was determined, and mice with a tumor size of 40–60 mm^3^ were enrolled for further study. Mice were intraperitoneally injected every 3 days with DMSO or T4O (40 mg/kg/day). In each group of nude mice, the length and width of the subcutaneous tumor were measured every 3 days using a vernier caliper, and the tumor volume was calculated as (mm^3^) = (length × width^2^)/2. The growth curve of the subcutaneously transplanted tumor was plotted according to the tumor volume. After 25 days of treatment, the nude mice in each group were sacrificed, the tumor tissue was removed, and the tumor weight was measured.

### 4.6. Measurement of Cellular Ferroptosis Levels

An assay kit for glutathione concentration was used (Solarbio, Beijing, China) according to the manufacturer’s instructions. MDA concentrations were determined using a Solarbio lipid peroxidation assay kit (Beijing, China) according to manufacturer’s instructions. A solarbio iron assay kit (Beijing, China) was used to measure iron concentrations according to the manufacturer’s instructions. An assay kit (GPXs Assay Kit) was used to measure relative GPX activity (Solarbio, Beijing, China).

### 4.7. RNA Sequencing

Total RNA was extracted from samples using the TRIzol reagent (Thermo Fisher Scientific, Waltham, MA, USA) provided by ThermoFisher. We constructed sequencing libraries from RNA samples whose RIN number was greater than 7.0, as determined by Bioanalyzer 2100 and RNA 6000 Nano LabChip Kit (Agilent, Santa Clara, CA, USA). Reverse transcription was performed using SuperScriptTM II Reverse Transcriptase (Invitrogen, Carlsbad, CA, USA), and mRNA was fragmented into short fragments following purification. The cDNA inserts averaged 300 ± 50 bp for the final library. Following the vendor’s protocol, we sequenced the cDNA using Illumina NovaseqTM 6000 (LC-Bio Technology, Ltd., Hangzhou, China). The count data were analyzed using the EdgeR package after high-quality clean reads were obtained and batch normalization was performed. *p* < 0.05 and |LogFC| ≥ 2 were used as test criteria for differentially expressed genes.

### 4.8. GEPIA Data Analysis

JUN expression in gliomas and adjacent tissues was retrieved and analyzed using the GEPIA database [36]. |LogChange| > 1 and *p* < 0.05 were set as significance thresholds. In order to differentiate between the low and high expression levels of genes in glioma tissues, median gene expression values were used as cut-off values. An analysis of the association between JUN mRNA expression and patient prognosis was performed using Kaplan–Meier curves.

### 4.9. Statistical Analysis

An analysis of all results was conducted using SPSS software (version 19.0). Student’s *t*-test, one-way analysis of variance, and least significant difference *t*-test were used to analyze differences between two and multiple groups. The acceptable threshold for significance was set at *p* < 0.05.

## 5. Conclusions

T4O treatment causes JUN downregulation, thus leading to the inhibition of glioma cell proliferation and induction of ferroptosis.

## Data Availability

Not applicable.

## Sample Availability

Samples of the compounds are available from the authors.

## Abbreviations

T4O	Terpinen-4-ol
JUN	JUN proto-oncogene
ROS	Reactive oxygen species
MDA	Malondialdehyde
GSH	Glutathione
GPX4	Glutathione peroxidase 4
RT-qPCR	Real-time quantitative polymerase chain reaction
